# Monsoon and continent: a climate-tectonic coupling system

**DOI:** 10.1093/nsr/nwag540

**Published:** 2026-08-26

**Authors:** Yongyun Hu

**Affiliations:** Department of Atmospheric and Oceanic Sciences, School of Physics, Peking University, China

Monsoons are one of the most important components of the climate system. They influence the lives of half of the people on Earth [1]. Monsoon circulation is driven by land–sea thermal contrast. In summer, surface winds carry moist air toward land. Convections over the land lift the moist air upward, generate precipitation, and release latent heat, which further enhances the monsoonal circulation. In winter, winds blow from land to ocean, causing the dry season.

Continents have undergone super-cycles between assembled supercontinents and fragmented plates. Pangea is the latest supercontinent, and it broke up in the early Jurassic. Continents were most dispersive in the late Cretaceous and then reassembled in the Cenozoic. Since monsoons are driven by land–sea thermal contrasts, these tectonic changes must play fundamental roles in shaping the monsoon system.

One of the most significant tectonic events in the Cenozoic was the collision between the Indian and Asian continents. It led to the formation of the Qinghai-Xizang Plateau, which largely shaped the South and East Asian monsoon. Despite extensive studies, the tectonic processes of the India–Asia collision remain debated. Yuan *et al.* [2] review five existing collision models and highlight three particularly contentious issues. They emphasize that the available evidence aligns with the triple-stage collision model involving the North India Sea. It is expected that the collision model will continue to be refined through the integration of multidisciplinary evidence and cross-disciplinary validation.

The simulations by Farnsworth *et al.* [3] demonstrate how the monsoon system is sensitive to tectonics. They perform three sets of simulations to examine influences of three independent paleogeographic reconstructions on the East Asian summer monsoon (EASM) in the past 145 million years. It is found that the strengthening of the Cenozoic EASM in the three sets of simulations all align with Tibetan and regional mountain orogeny, but the timing and intensity of EASM development are markedly diverse. They also show that the Cretaceous EASM is sensitive to remote continental configurations.

Over tectonic timescales, monsoons are governed by land–sea distributions, not by CO_2_ concentrations [4]. However, monsoons are sensitive to CO_2_ over shorter timescales, such as the orbital timescale. As tectonics cause changes in atmospheric CO_2_, through the carbon cycle between Earth’s interior and the surface, it is important to quantify the carbon cycle. Zhao *et al.* [5] propose a novel two-phase flow dynamics model to reconstruct volcanic eruption processes in a mantle-plume–lithosphere setting. They show that the contrast between low and high viscosity components determines the frequency of volcanic eruptions, and that the power-law relationship between them is the key signature of phase transitions in two-phase flow systems.

Another important tectonic process in the Cenozoic was the northward movement of the Australia plate, which separated from the Antarctic ∼40 million years ago and gradually moved to the present location. Early studies suggested that the Australian-Indonesian Monsoon (AIM) formed right after Australia’s separation from the Antarctic. However, recent evidence and simulations argue that the AIM formed much later [6]. Mohtadi *et al.* [6] also examined AIM variabilities over orbital to interannual timescales.

The coupled monsoon-continent system and its evolution have important implications for the living environment of human beings. Its seasonal variations between wet and dry conditions are also important for ecosystems and formations of exogenetic ores. Thus, further research requires systematic and interdisciplinary studies.
